# Post-traumatic Stress Symptoms in Adolescents Exposed to the Earthquake in Lombok, Indonesia: Prevalence and Association With Maladaptive Trauma-Related Cognition and Resilience

**DOI:** 10.3389/fpsyt.2021.680393

**Published:** 2021-11-08

**Authors:** Suzaily Wahab, Li Ling Yong, Wei Keong Chieng, Myristica Yamil, Noor Azah Sawal, Nurul Qiyaam Abdullah, Cyntiya Rahmawati Muhdisin Noor, Siti Mardiyah Wd Wiredarma, Rosnah Ismail, Aisya Hanim Othman, Hanafi Ahmad Damanhuri

**Affiliations:** ^1^Department of Psychiatry, Universiti Kebangsaan Malaysia Medical Centre, Wilayah Persekutuan Kuala Lumpur, Malaysia; ^2^Faculty of Health Science, Universitas Muhammadiyah Mataram, Kota Mataram, Indonesia; ^3^Department of Community Health, Universiti Kebangsaan Malaysia Medical Centre, Wilayah Persekutuan Kuala Lumpur, Malaysia; ^4^Department of Biochemistry, Faculty of Medicine, The National University of Malaysia, Wilayah Persekutuan Kuala Lumpur, Malaysia

**Keywords:** natural disasters, post-traumatic stress, cognition, resilience, adolescent

## Abstract

**Background:** Natural disasters may physically and psychologically affect individuals and their surrounding community. This study determines the prevalence of post-traumatic stress (PTS) symptoms and its association with maladaptive trauma-related cognition and resilience among adolescents post-earthquake.

**Materials and Methods:** Data were collected, in this cross-sectional study, during an intervention program post-earthquake held in a state high school located at Lombok, Indonesia. The study sample engaged students 14–19 years of age using the purposive sampling method. The questionnaires used to measure PTS symptoms, maladaptive trauma-related cognition, and resilience were Children's Revised Impact of Event Scale-13 (CRIES-13), Child Post-Traumatic Cognitions Inventory (CPTCI), and Child and Youth Resilience Measure-Revised (CYRM-R), respectively.

**Results:** The prevalence of PTS symptoms was 69.9%. Among the respondents, 61.37% were female and 56.48% had mothers with lower educational levels. Using multiple linear regression, the final predictors of PTS symptoms were excessive reactions (e.g., wailing loudly, miserable shrieking) of proxy during earthquake (*β* = 3.283, *p* = 0.005), maladaptive trauma-related cognition (*β* = 0.224, *p* = 0.002), and resilience (*β* = 0.192, *p* < 0.001) with female gender (*β* = 7.350, *p* < 0.001) as a control variable. Through simple linear regression, victims who witnessed injury or death during the earthquake (*p* = 0.003), had a proxy died during the earthquake (*p* = 0.01), and trapped victims or those who had difficulty escaping (*p* = 0.01) were identified to potentially predict the occurrence of PTS symptoms, warranting further study.

**Conclusion:** The presence of excessive proxy reactions during the earthquake, maladaptive trauma-related cognition, and resilience in adolescents exposed to a natural disaster are worth targeting and prioritizing in future post-disaster interventions.

## Introduction

Natural disasters have physical and psychological impacts on individuals and their surrounding community. The psychological effects of such catastrophes on a person include depression, anxiety, and stress-related illnesses (1). Post-traumatic stress disorder (PTSD) is one of the stress-related disorders that may develop following exposure to a major traumatic event. PTSD sufferers experience episodes of recurrent intrusion symptoms such as distressing memories of traumatic events, nightmares, and dissociative reactions, as well as avoidance, negative changes in cognition and mood, and hyperarousal (2).

The risk factors for the development of PTSD include extreme age (children, adolescents, and elderly), female gender, low socioeconomic status, low level of education, and aspects of trauma severity (direct and indirect exposure to disaster, witnessing destruction of property, worry about others, witnessing death and injuries of others and physical injury to self during an event of a natural disaster) (3, 4). A study in Indonesia by Marthoenis et al. (5) found that 58.3% of adolescents showed clinical symptoms of PTSD 6 months after the 2016 Aceh Earthquake. The high prevalence of post-traumatic stress (PTS) symptoms in adolescents is worrying as this may affect their daily functioning and cognition. The worse scenario was PTS symptoms that persisted until adulthood resulting in the need for PTSD treatment secondary to childhood trauma (6). Maladaptive cognitive processes experienced during traumatic events may contribute to the long-lasting PTS symptoms. Meiser-Stedman et al. (7) observed that cognitive processes play crucial in the development and persistence of PTS symptoms in both children and adolescents. In the study, all the perceived threats, overwhelming feelings, and confusion during the traumatic event contributed to the onset of acute PTS symptoms. Poorly elaborated, sensory-based memories, dissociation, rumination, and negative appraisals were closely associated with persistent PTS symptoms.

A recent cohort study conducted by Altamore et al. (8) postulated that repeated exposure to earthquakes increased the prevalence of developing psychological distress and PTSD, in which 0.5% prevalence of probable PTSD assessed in 2015 spiked to 16.9% in 2017 after the second earthquake. Although the cohort samples experienced a low level of distress for 8 years after the first earthquake, a second exposure to the earthquake had led to a higher level of psychological and PTS symptoms. Even after the natural disaster has subsided, visiting or being at the place of incidence may still trigger trauma and negative memories that can affect a person's mental and physical health (9). Therefore, the frequency of exposure to earthquakes may influence the development of PTSD and the resilience of adolescents exposed to natural disasters.

Also, resilience is fundamental for people to positively function after a disaster. Resilience was strongly associated with reduced PTS symptoms 3 months after a hurricane (10) and improved quality of life after earthquake exposure (11). Moreover, Smith et al. (12) revealed that a high level of resilience was associated with lower probabilities of developing mental health issues such as depression, anxiety, and stress. Adolescents who experienced the 2016 Fort McMurray wildfire also showed that those who had high resilience showed higher self-esteem and quality of life scores (13).

Several factors that increased resilience in the post-disaster population included self-efficacy, having coping strategies, and good social support (14). Positive thinking and self-motivation were among the strategies used in self-efficacy by the population. Coping strategies such as turning to God and religious activities had helped them to remain hopeful in adversities. Social support given by government and non-government organizations also proved to be helpful in building resilience in the population (14).

Developing countries have started integrating mental health and psychosocial support in their disaster preparedness and response (15–17). However, recent studies showed that mental health problems especially PTSD, depression, and anxiety were still relatively high in these countries (18, 19). Adolescents are vulnerable to their surroundings and may not have adequate knowledge of how to cope with mental health issues and what to do in response to natural disasters. There are limited studies on the effects of repeated trauma exposures on adolescents' mental health. If no prompt identification and treatment, PTSD may persist for a long period of time and impair the children's or adolescents' function and development (20, 21). Therefore, this study aimed to determine the relationship between PTS symptoms, maladaptive trauma-related cognition, and resilience in adolescents exposed to the earthquakes in Lombok, Indonesia.

## Materials and Methods

This cross-sectional study was part of an intervention under a student mobility program conducted by the Student Council of the National University of Malaysia in a state high school located at Lombok, Indonesia from July 15 to 21, 2019. The aim of the program was to provide humanitarian aid and psychosocial support, including interventions such as relaxation and coping techniques, for the local adolescents exposed to multiple earthquakes. Lombok is well known as a disaster-prone area due to the island's location in the Pacific Ring of Fire characterized by active movement of tectonic plates resulting in frequent natural disasters. Multiple earthquakes occurred within 9 months: a strong magnitude earthquake (6.4) on July 29, 2018, followed by a major earthquake of 7.0 magnitude on August 5, 2018, and a moderate magnitude earthquake (5.8) on March 17, 2019. The current study involved a group of adolescents in Lombok who survived the earthquakes.

### Description of Primary Data

**Figure 1:**
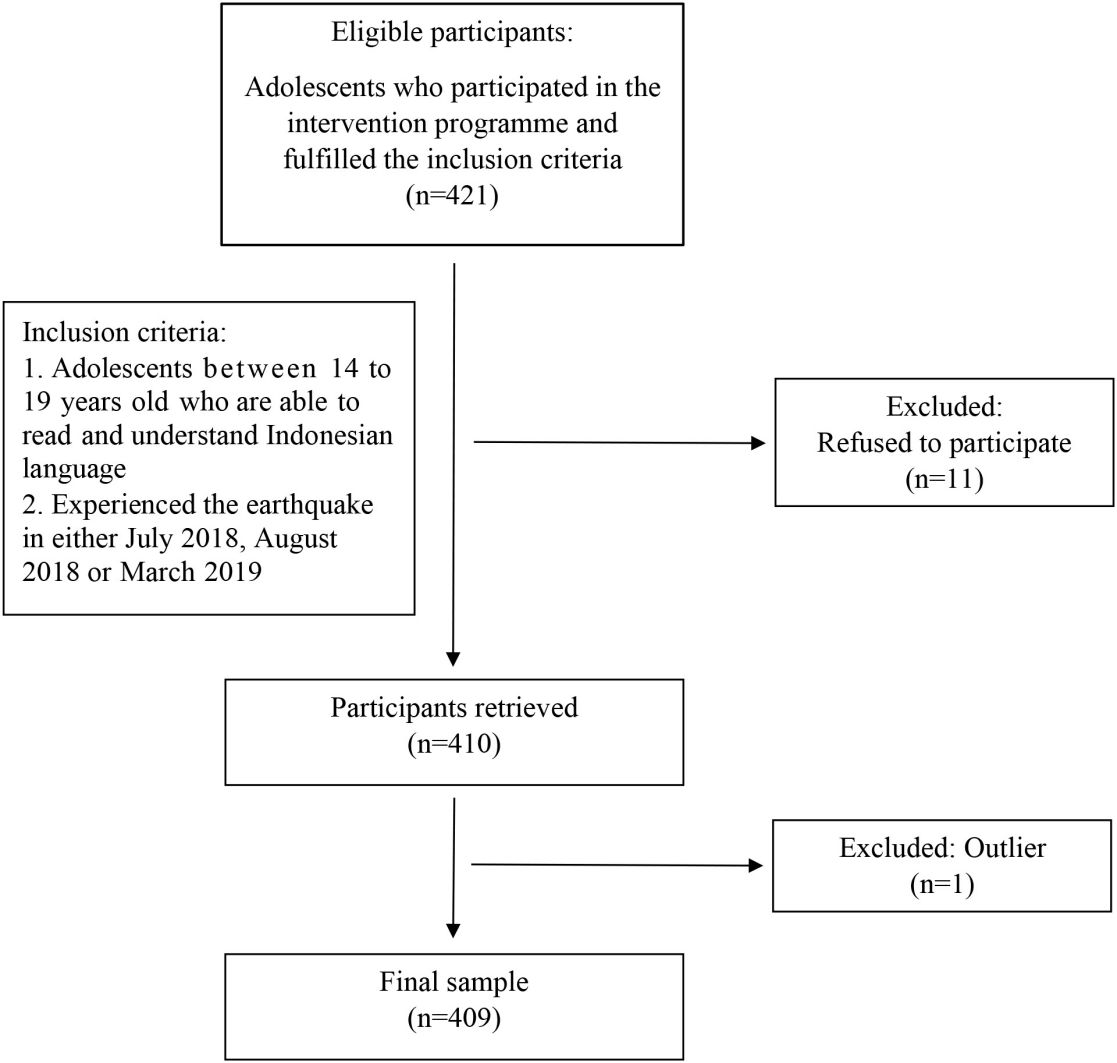
Sampling process flowchart.

Researchers collected data from secondary school students who participated in the intervention program by purposive sampling method. The inclusion criteria were adolescents ranging from 14 to 19 years old with the ability to read and understand Indonesian language and had experienced at least one of the earthquakes in the 9 months. The study used the WHO definition of adolescents as those who fall within the age range of 10–19 years (22). Exclusion criteria applied to adolescents whose parents did not give consent or those who were unable to understand the questionnaire even under the guidance of the facilitators. The WHO sample size formula (23) yielded a calculated sample size of 246 with the absolute precision of 5% and the level of significance of 95%. The study obtained 421 participants who fulfilled the inclusion criteria and were enrolled for survey completion before the intervention program. However, 11 adolescents refused to participate in the study which resulted in a total sample of 410 participants (Figure 1). The assembly point of the school was the data collection location. Thirty facilitators divided the participants into small groups of 15, and participants were given self-rated questionnaires to be answered. Indonesian facilitators were readily available to assist with further inquiries on any of the questions.

### Instruments

The back-translated questionnaires in Indonesian language were used in this study, including socio-demographic and disaster correlates, Child Revised Impact of Events Scale-13 (CRIES-13) (24), Child's Post-Traumatic Cognitions Inventory (CPTCI) (25), and Child and Youth Resilience Measure-Revised (CYRM-R) (26).

### Sociodemographic and Disaster Correlates

The sociodemographic questionnaire measured age, gender, household numbers, and educational level of participants and parents. We reviewed disaster correlates, including the reaction of parents during the incident, frequency of exposure to the earthquake, destruction of property or residence, recent move to a new school or place, trapped or separated from proxy during the event, and experienced or witnessed injury and death during the incidents. This questionnaire described the background of the study population.

### CRIES-13

CRIES-13 is a screening instrument to assess children at risk of PTSD. It consists of 13 items measuring intrusion (4 items), avoidance (4 items), and arousal (5 items) with a cut-off point of 30. A score of 30 and above indicates the positive screening of PTSD. The scoring for each item uses 0 (Not at all), 1 (Rarely), 3 (Sometimes), and 5 (Often), making the total score ranges from 0 to 65. Cronbach's alpha (27) was found to be 0.80 for the total scale and was 0.70, 0.73, and 0.60 for intrusion, avoidance, and arousal subscales, respectively. Perrin et al. (28) had examined the specificity and sensitivity of the scale in two different samples which included 1) clinical samples comprising children and adolescents (7–18 years old) in Child Traumatic Stress Clinic who were being referred for PTSD assessment, and 2) accident and emergency room samples that included victims of road traffic accidents or assault cases aged from 10 to 16 years. Both samples showed good sensitivity and specificity with a cut-off point of 30 given that the clinical sample reported sensitivity of 0.91 and specificity of 0.65 while the accident and emergency sample reported sensitivity of 0.86 and specificity of 0.73.

### CPTCI

Maladaptive trauma-related cognition is assessed using CPTCI consisting of 25 items with two sub-scales: 1) permanent and disturbing change (13 items), and 2) fragile person in a scary world (12 items). A 4-point range of responses from do not agree at all (1) to agree a lot (4) is used to rate each item. The total possible score ranges from 25 to 100, with higher scores corresponding to more negative trauma-related cognition. The reported Cronbach's alpha for this scale ranged from 0.86 to 0.93 (25).

### CYRM-R

CYRM-R consists of 17 items that measure personal resilience (10 items) and caregiver resilience (7 items). A 5-point Likert scale, ranging from not at all (1) to a lot (5), rates each item. The total score ranges from 17 to 85. Cronbach's alpha was 0.902 for both personal and caregiver resilience (26, 29).

### Ethical Issues

The study obtained approval from the institution's ethics committee. Written consent was obtained from the parents of the participants prior to study enrollment. Participants who showed high post-traumatic stress symptoms were advised to seek psychological treatment at the nearby hospital.

### Statistical Analysis

Multiple imputations replaced the missing values in the data. We used descriptive analysis to describe the basic demographic data and earthquake exposure among the participants. Student *t*-test and ANOVA were both used to outline the relationship between significant demographic data shown in previous studies, trauma experience prior to or preceding the incidents and frequency of earthquake exposure to the development of PTS symptoms, resilience, and trauma-related cognition of the participants. Pearson's correlation described the strength of the relationship between an interval variable with another interval variable, while point biserial correlation was used for the relationship between an interval variable with another nominal variable. Multiple linear regression determined the predictors of post-traumatic stress symptoms.

## Results

The final participants included were 409 with 158 (38.6%) males and 251 (61.4%) females. One subject was removed from the study as it was an outlier for the CPTCI questionnaire, identified with scatter plot and interquartile range method. A suggested cut-off of 30 was used and the prevalence of probable PTSD was 69.9% for the CRIES questionnaire. The proportion of exposure to the earthquake at least once, twice, and three times were 21.3, 28.4, and 50.4%, respectively. Table 1 presents the questionnaire responses.

**Table 1:** Descriptive statistics of all variables.

**Variables** **(***n*** = 409)**	**M ± SD**	**N**	**%**
**A. Sociodemographic variables**			
1. Age	16.24 ± 0.98	–	–
2. Female gender	–	251	61.37
3. Currently living with parents	–	381	93.15
4. For the past 1 year, living with parents	–	376	91.93
5. Household fewer than 4 members	–	70	17.11
6. Low paternal education level	–	170	41.56
7. Low maternal education level	–	231	56.48
**B. Variables of earthquake experience**			
1. Exposed to earthquake July 2018	–	330	80.68
2. Exposed to earthquake August 2018	–	387	94.62
3. Exposed to earthquake March 2019	–	220	53.79
4. Exposed to earthquake >1 time	–	322	78.73
5. Injured during earthquake	–	85	20.78
6. Witnessed injury or death during earthquake	–	257	62.84
7. Proxy injured during earthquake	–	160	39.12
8. Proxy died during earthquake	–	127	31.05
9. Separated from proxy during earthquake	–	67	16.38
10. Excessive response of proxy	–	200	48.90
11. Destruction of residence	–	317	77.51
12. Destruction of property	–	191	46.70
13. Trapped or difficult escape	–	69	16.87
14. Parents lost occupation	–	60	14.67
15. Move to a new school	–	16	3.91
16. Move to a new place	–	234	57.21
**C. Variables of trauma experience**			
1. Experienced serious accidents before earthquake	–	61	14.91
2. Experienced other natural disasters before earthquake	–	44	10.76
3. Experienced man-made disasters before earthquake	–	60	14.67
4. Experienced serious injuries or near-death situation before earthquake	–	23	5.62
5. Witnessed death or serious injuries before earthquake	–	65	15.89
6. Robbery victim before earthquake	–	12	2.93
7. Beaten until injured before earthquake	–	17	4.16
8. Trauma exposure before earthquake	–	153	37.41
9. Lived in shelter home after earthquake	–	350	85.57
10. Lived in shelter home for ≥6 months	–	148	36.19
**D. Main variables**			
1. PTS symptoms (score ≥ 30, PTS symptoms)	36.54 ± 12.53	286	69.93
2. Resilience (high level)	68.04 ± 10.89	149	36.43
3. Maladaptive trauma-related cognition (score > 46, pessimistic)	49.86 ± 7.95	293	71.64

*PTS, post-traumatic stress*.

As predicted, results of independent *t*-test showed a significant difference in the PTS symptoms across gender, in which females [*M* = 39.99, SD = 11.46, *t*_(407)_ = −7.48, *p* < 0.001] had higher PTSD scores than males. Besides, adolescents with mothers of lower educational level [*M* = 37.56, SD = 12.42, *t*_(399)_ = 2.02, *p* = 0.04] scored significantly higher in PTS symptoms; in comparison with adolescents whose mother had education of at least secondary level. There were also significantly higher PTS scores among adolescents who had witnessed injury or death [*M* = 37.96, SD = 12.36, *t*_(407)_ = −3.02, *p* = 0.003] compared with those who did not; and among adolescents who had family, guardian, or acquaintance who died during earthquake [*M* = 38.87, SD = 13.07, *t*_(407)_ = −2.55, *p* = 0.01] in comparison with those who did not have deceased proxy during earthquake. In addition, PTS scores were significantly higher among adolescents whose parents reacted excessively during earthquake (e.g., loud wailing, loud prayers) [*M* = 39.23, SD = 12.24, *t*_(407)_ = −4.33, *p* ≤ 0.001], in contrast to those whose parents did not react this way. In comparison with the differences in PTS scores, adolescents who were trapped or experienced difficult escape during earthquake [*M* = 39.90, SD = 11.95, *t*_(407)_ = −2.46, *p* = 0.01] scored significantly higher than those who did not experience this. The adolescents who have higher level of resilience were found to have higher PTS scores [*M* = 38.71, SD = 12.34, *t*_(407)_ = −2.69, *p* = 0.01] than those with lower level of resilience. Details are tabulated in Table 2.

**Table 2:** Mean PTS score differences between groups.

**Variables** **(***n*** = 409)**		**PTS score,M ± SD**	***t-*** **value**	***P*** **-value**
**A. Sociodemographic variables**				
1. Gender	Male	31.05 ± 12.24	−7.48	<0.001^*^
	Female	39.99 ± 11.46		
2. Currently living with both parents/guardian	Parents	36.83 ± 12.16	1.34	0.19
	Guardian	32.54 ± 16.59		
3. For the past 1 year, living with both parents/guardian	Parents	36.78 ± 12.13	0.83	0.41
	Guardian	34.31 ± 16.40		
4. Household size	Small	35.64 ± 13.24	−0.70	0.49
	Big	36.79 ± 12.38		
5. Father education level	Low	37.61 ± 12.57	1.43	0.15
	High	35.79 ± 12.55		
6. Mother education level	Low	37.56 ± 12.42	2.02	0.04^*^
	High	35.01 ± 12.63		
**B. Variables of earthquake experience**				
1. Exposure to earthquake July 2018	No	36.91 ± 12.99	0.30	0.77
	Yes	36.45 ± 12.44		
2. Exposure to earthquake August 2018	No	32.36 ± 16.86	−1.21	0.24
	Yes	36.78 ± 12.23		
3. Exposure to earthquake March 2019	No	36.03 ± 12.69	−0.77	0.45
	Yes	36.98 ± 12.41		
4. Frequency of exposure to earthquake (1 time/more than 1)	1 time	34.72 ± 13.94	−1.52	0.13
	>1 time	37.03 ± 12.10		
5. Injured during earthquake	No	36.08 ± 12.25	−1.44	0.15
	Yes	38.28 ± 13.50		
6. Witness injury or death during earthquake	No	34.13 ± 12.49	−3.02	0.003^*^
	Yes	37.96 ± 12.36		
7. Proxy injured during earthquake	No	35.69 ± 12.24	−1.72	0.09
	Yes	37.86 ± 12.91		
8. Proxy died during earthquake	No	35.49 ± 12.16	−2.55	0.01^*^
	Yes	38.87 ± 13.07		
9. Separated from proxy during earthquake	No	36.32 ± 12.33	−0.78	0.44
	Yes	37.63 ± 13.56		
10. Excessive response of proxy	No	33.97 ± 12.30	−4.33	<0.001^*^
	Yes	39.23 ± 12.24		
11. Destruction of residence	No	35.45 ± 12.48	−0.95	0.34
	Yes	36.85 ± 12.55		
12. Destruction of property	No	35.65 ± 12.27	−1.54	0.13
	Yes	37.55 ± 12.78		
13. Trapped or difficult escape	No	35.86 ± 12.56	−2.46	0.01^*^
	Yes	39.90 ± 11.95		
14. Parents lost occupation	No	36.46 ± 12.54	−0.30	0.77
	Yes	36.98 ± 12.61		
15. Move to a new school	No	36.74 ± 12.40	1.64	0.10
	Yes	31.50 ± 15.13		
16. Move to a new place	No	35.43 ± 13.28	−1.54	0.12
	Yes	37.36 ± 11.91		
**C. Variables of trauma experience**				
1. Experienced serious accidents before earthquake	No	36.56 ± 12.48	0.10	0.92
	Yes	36.39 ± 12.95		
2. Experienced other natural disasters before earthquake	No	36.63 ± 12.57	0.43	0.67
	Yes	35.77 ± 12.34		
3. Experienced man-made disasters before earthquake	No	36.32 ± 12.78	−0.87	0.39
	Yes	37.83 ± 11.03		
4. Experienced serious injuries or near-death situation before earthquake	No	36.50 ± 12.59	−0.23	0.82
	Yes	37.13 ± 11.80		
5. Witnessed death or serious injuries before earthquake	No	36.22 ± 12.74	−1.18	0.24
	Yes	38.22 ± 11.31		
6. Robbery victim before earthquake	No	36.48 ± 12.47	−0.57	0.57
	Yes	38.58 ± 15.10		
7. Beaten until injured before earthquake	No	36.78 ± 12.38	1.85	0.07
	Yes	31.06 ± 15.06		
8. Trauma exposure before earthquake	No	36.61 ± 12.58	0.15	0.88
	Yes	36.42 ± 12.49		
9. Lived in shelter home after earthquake	No	34.44 ± 13.92	−1.39	0.17
	Yes	36.89 ± 12.27		
10. Duration of stay	Short	37.22 ± 12.51	1.44	0.15
	Long	35.32 ± 12.68		
**D. Main variables**				
1. Resilience	Low	35.28 ± 12.50	−2.69	0.01^*^
	High	38.71 ± 12.34		
2. Maladaptive trauma-related cognition	Positive	37.10 ± 11.94	1.35	0.18
	Negative	35.12 ± 13.88		

*PTS, post-traumatic stress*.

* *Indicate statistically significant p < 0.05*.

The results of zero-order correlation are shown in Table 3. This bivariate analysis was primarily used to guide the selection of variables into the regression model. Findings show that when compared with males, females had a positive but weak PTS score (r_pb_ = 0.35). Maternal education level had a negative but negligible correlation with PTS symptoms (r_pb_ = −0.10). In contrast, maladaptive trauma-related cognition (higher score indicating more negative trauma-related cognition) and resilience had statistically significant weak, positive correlation with PTS symptoms (*r* = 0.12 and *r* = 0.21). In addition, the variables of trauma exposure listed in Table 3 also correlated positively and significantly with PTS symptoms (*r* = 0.12–0.21), indicating weak relationships.

**Table 3:** Matrix of inter-variable relationships.

	**1**	**2**	**3**	**4**	**5**	**6**	**7**	**8**	**9**	**10**
1. Age	–									
2. Gender	−0.02	–								
3. Mother education level	−0.07	−0.08	–							
4. Witnessed death/injury	0.14^**^	0.10	0.02	–						
5. Proxy died	0.04	0.12^*^	0.02	0.30^**^	–					
6. Excessive reaction	0.03	0.19^**^	−0.02	0.20^**^	0.24^**^	–				
7. Trapped/difficult escape	−0.04	0.18^**^	0.10	0.14^**^	0.05	0.13^**^	–			
8. Resilience	0.11^*^	0.23^**^	−0.05	0.07	0.11^*^	0.04	0.02	–		
9. Maladaptive trauma-related cognition	−0.02	−0.02	−0.08	0.03	−0.09	0.11^*^	0.22^**^	−0.19^**^	–	
10. PTS symptoms	0.10	0.35^**^	−0.10^*^	0.15^**^	0.13^*^	0.21^**^	0.12^*^	0.21^**^	0.12*	–

*PTS, post-traumatic stress*.

** *p <0.01 and*

* *p <0.05*.

According to the regression analysis results (Table 4), the model explained 17% of the variance and was a significant predictor of PTS symptoms [*F*_(4,404)_ = 22.06, *p* ≤ 0.001]. The female gender contributed most significantly to the model (*β* = 7.350, *p* < 0.001), followed by excessive reaction of parents during earthquake (*β* = 3.283, *p* = 0.005), maladaptive trauma-related cognition (*β* = 0.224, *p* = 0.002), and resilience (*β* = 0.192, *p* < 0.001). Simple linear regression demonstrated that the statistical significance of adolescents who witnessed injury or death during earthquake (*p* = 0.003), had a proxy died during earthquake (*p* = 0.01), and those who were trapped or had a difficult escape (*p* = 0.01) may potentially predict the occurrence of PTS symptoms.

**Table 4:** Predictors of PTS symptoms.

**Variable**	**SLR^a^**			**MLR ^b^**			
	**β^c^**	**(95% CI)**	***P*** **-value**	**β^d^**	**(95%CI)**	**t-stat**	***p*** **-value**
Age	1.223	(−0.015, 2.461)	0.053	–	–	–	–
Female gender	8.941	(6.593, 11.290)	<0.001^**^	7.350	(4.964, 9.736)	6.06	<0.001^**^
Excessive reaction	5.258	(2.873, 7.644)	<0.001^**^	3.283	(1.004, 5.562)	2.83	0.005^**^
Maladaptive trauma-related cognition	0.189	(0.037, 0.342)	0.015^*^	0.224	(0.080, 0.367)	3.07	0.002^**^
Resilience	0.244	(0.134, 0.353)	<0.001^**^	0.192	(0.085, 0.299)	3.53	<0.001^**^

a *Simple linear regression*.

b *Multiple linear regression (adjusted R^2^ = 17%). The model reasonably fits well, model assumption was met; there is no interaction between independent variables and dependent variable, and no multicollinearity problem*.

c *Crude regression coefficient*.

d *Adjusted regression coefficient*.

* *Indicate statistically significant p < 0.05*.

** *Indicate statistically significant p < 0.01*.

## Discussion

The prevalence of PTS symptoms among adolescents post-earthquake was 69.9% in this study, which was higher compared with other studies. The prevalence of PTSD among adolescents 6 months after Aceh earthquake and 12 months post-Wenchuan earthquake were 47 and 17.5%, respectively (5, 30). Furthermore, Rahmadian et al. (31) reported that 19.9% of children and adolescents who were survivors of natural disasters in Indonesia fulfilled the criteria of PTSD diagnosis. The prevalence may be affected by differences in the study population, method of assessments, time-lapse after earthquake, and the severity of the event. A few factors may contribute to the high prevalence of PTS symptoms in this study. Older children were found to be more susceptible to the development of PTSD compared with younger children (32). Other than that, recurrent exposures to disasters can potentially bring a negative additive effect on mental health (33). Moreover, the purposive sampling method of this study may also contribute to the high prevalence.

This study found a significant difference between gender with regards to severity of PTS symptoms. According to the study results, female adolescents had higher PTS symptoms score compared with males. This result is consistent with the meta-analysis by Furr et al. (34) that suggested females are more vulnerable to the development of PTS symptoms. Derivois et al. (35) and Cadichon et al. (36) also concluded that female gender is an important PTSD risk factor among the study's population. Several explanations including stronger apprehension of threat, higher extent of peritraumatic dissociation, poor social support, and gender differences in acute subjective responses to trauma were suggested by a research review (37). Witnessing injury or death during earthquakes and excessive response from parents during and after earthquakes were significantly associated with higher PTS symptoms scores. Kilic et al. (38) reported that negative effect on children was more significant when fathers became more irritable and detached. Ekşi et al. (39) and Ma et al. (40) also reported that witnessing death and suffering as well as extreme reactions from parents or other adults during and following earthquake are significant risk factors for the development of PTS symptoms. These reactions include wailing, crying, and praying loudly, which may raise children's fright, insecurity, and helplessness. As parents or adults are coping models, the children may perceive the event as frightening or even threatening after observing the adults' reactions. This finding suggests that the subjective element of disaster experience contributes to an adolescent's post-trauma response, which should be made aware and recognized in psychological interventions.

In addition, maladaptive trauma-related cognitions measured by CPTCI was found to be one of the predictors that was positively associated with PTS symptoms. A meta-analysis showed cognitive deficits regarding working memory, executive function, verbal learning and memory, visual learning and memory, information processing speed, and visuospatial function play important roles in PTS symptoms and are associated with the disorder (41). Stallard et al. (42) supported that negative trauma appraisal and cognitive coping style lead to permanent PTS symptoms. This is because the ongoing negative trauma appraisal contributes as a factor that causes the traumatic event to be experienced as a current threat even though the traumatic event happened in the past. A significant difference in cognitive style between adolescents with high risk of PTSD and low risk of PTSD was also reported in research conducted following the Wenchuan earthquake in China (40). Thus, adolescents with negative and maladaptive trauma-related cognitions require careful screening and monitoring for PTSD development.

The findings of the study revealed that resilience was significantly and positively associated with PTS symptoms among adolescents exposed to Lombok earthquakes. A study done among street children in Haiti showed that those with severe PTS symptoms possessed higher level of resilience (43). On the contrary, other studies showed adolescents with higher resilience level had lower risk of developing PTS symptoms (4, 44–46). Resilience was also shown to be a predictor of PTSD in a recent study (47). While resilience has been an established protective factor that modulates response toward trauma, the study results showed that adolescents with higher resilience level were significantly associated with higher PTS scores. We postulated that the high scoring of PTS symptoms in these participants may be contributed by repeated exposures to earthquakes within a 9-month period. In this study, as high as 50.4% of participants experienced earthquakes three times in Lombok, Indonesia. Previous studies have observed dose gradients of trauma, in which more intense, more prolonged, and more cumulative traumas were associated with more symptoms (48, 49). Adolescents who experienced additional trauma after an earthquake exposure were also shown to be more likely to develop delayed or chronic PTS symptoms (50). Moreover, a longitudinal study over 17 years among child survivors of dam collapse found that PTSD remained present at a higher-than-normal rate; however, resilience and recovery were observed in most exposed individuals (51).

Also, resilience is not merely a personality trait, but it has been broadening to a more ecological context that can be developed at any age based on individual relationships and environments (52–54). It is conceivable that in the study, the measure of current resilience level captured both effects of existing resilience and growth following trauma exposures. The high level of resilience in this population may be contributed by various external resources including good social support from caregivers and community, religion, spirituality, and cultural resources, not solely associated with the experiences from repeated earthquakes. Different dimensions of social support from family members, peers, teachers, and community were identified as resources of resilience in previous studies (55–57). The study participants scored high with regards to relational resilience, which also suggested that family members and peers were important resources for adolescents to show resilience. Besides, our participants are all Muslims. While religion and spirituality were shown to promote resilience (58–60), a study conducted in Islamic population pointed out that religion was a key factor in facing the tragedy of tsunami in their communities and reinforcing social ties (61).

Several potential predictors of PTS symptoms were ascertained in this study, including adolescents who witnessed injury or death during earthquake, had a proxy died during earthquake, and those who were trapped or had a difficult escape. These exposures to traumatic events during earthquake were significantly associated with higher PTS scores, which were consistent with other studies (62–64). The results may imply that adolescents who had higher degree of physical exposure to a disaster portrayed a larger impact on their reactions or coping in post-trauma adjustment. However, these findings must be cautiously interpreted due to the weak association in the study results. The potential of these exposure indicators as predictors of PTS symptoms warrants future research to gain further insight.

More importantly, the study results demonstrated that there was a negative correlation between maladaptive trauma-related cognitions and resilience. Limited studies have adequately answered the relationship between these two salient indicators of PTS symptoms. This finding will add value to and consolidate the holistic interventions among adolescents exposed to disasters as cognitive functioning is one of many aspects that is often disregarded in post-traumatic psychopathology. Our growing understanding of negative trauma-related appraisals and resilience will hopefully help with the development of more strategies for PTSD management. The psychological therapy of PTSD is deemed to focus on the key role of various predictors in overcoming the adversity and trauma response among adolescents, for instance, avoidance, intrusion, and arousal. Intervention designs should be based on the unique gender differences in responding to traumatic events as females exhibited higher PTS scores compared with males. Also, it is necessary to be aware that proxy's reaction in adverse situations may influence children's stress responses. Thus, family-based therapy is recommended as the importance of proxy's response in preventing stress reactions of adolescents needs to be emphasized. Moreover, in the situation of family members who were affected during earthquakes, the vulnerable adolescents should be screened vigorously and provided intervention, if necessary, even though they appeared physically or mentally well.

This study has some limitations. A cross-sectional study design was used and hence, the relationship between the exposures and outcomes could not be well explained. Thus, further longitudinal studies on the same population may produce more valuable results. Besides, the collection of data took place a year after the earthquake incidents. The results may not explain the direct acute effects of the earthquakes and may not be applicable to other time frames after earthquakes. The cognition and resilience of the samples might be affected by other confounding variables such as social support in this period. Furthermore, the back-translated questionnaires in Indonesian language have not been validated in the local population. The questionnaires were administered by self-reporting method, which carried the risk of information bias and led to less accuracy. Future studies may employ other information sources such as caregivers' and teachers' reports, as well as clinical interviews to enhance the accuracy and reliability of the findings.

## Conclusions

The findings of this study revealed that the natural disaster, namely earthquakes, brought notable psychological impacts on adolescents in Lombok, Indonesia. This was discernible by the high prevalence of PTS symptoms in the participants. Several predictors of PTS symptoms among adolescents post-earthquakes were identified in this study, including maladaptive trauma-related cognitions, resilience, and excessive reactions of proxy during earthquakes. These are worth targeting and prioritizing in future post-disaster interventions. Adolescents who witnessed injury or death, had a proxy died, and had difficulty in escaping or being trapped during earthquakes were identified as the potential predictors of PTS symptoms, which warrant further studies. Early identification of vulnerable adolescents is essential to offer preventive measures and to provide effective psychological support if necessary. The negative association between maladaptive trauma-related cognition and resilience further emphasized the role of cognition in post-traumatic psychopathology and should be further investigated in future studies.

## Data Availability Statement

The datasets presented in this article are not readily available because data cannot be shared due to confidentiality issues. Requests to access the datasets should be directed to Suzaily Wahab, suzailywhb@yahoo.com.

## Ethics Statement

The study involving human participants was reviewed and approved by Secretariat of Research and Innovation, Faculty of Medicine, The National University of Malaysia and Ethical Committee of Medical Research, Faculty of Medicine, The University of Mataram. Written informed consent to participate in this study was provided by the participants' legal guardian/next of kin.

## Author Contributions

SW and HD conceptualized and contributed to the study design. LY, WC, MY, NS, NA, CM, and SMW collected the data. RI, HD, WC, LY, and AO performed the data analysis and interpretation. SW, LY, WC, MY, NS, and HD wrote the draft of the article. Finally, SW, RI, and HD critically revised the article. All authors have made substantive intellectual contributions to the manuscript, read, and approved the final article.

## Funding

This is an independent research funded and supported by the Ministry of Higher Education, Malaysia and Universiti Kebangsaan Malaysia Medical Centre.

## Conflict of Interest

The authors declare that the research was conducted in the absence of any commercial or financial relationships that could be construed as a potential conflict of interest. The reviewer CN declared a shared affiliation, with no collaboration, with the authors SW, LY, WC, MY, NS, RI, AO, and HD at the time of the review.

## Publisher's Note

All claims expressed in this article are solely those of the authors and do not necessarily represent those of their affiliated organizations, or those of the publisher, the editors and the reviewers. Any product that may be evaluated in this article, or claim that may be made by its manufacturer, is not guaranteed or endorsed by the publisher.
